# Ultrasound-on-chip platform for medical imaging, analysis, and collective intelligence

**DOI:** 10.1073/pnas.2019339118

**Published:** 2021-07-01

**Authors:** Jonathan M. Rothberg, Tyler S. Ralston, Alex G. Rothberg, John Martin, Jaime S. Zahorian, Susan A. Alie, Nevada J. Sanchez, Kailiang Chen, Chao Chen, Karl Thiele, David Grosjean, Jungwook Yang, Liewei Bao, Rob Schneider, Sebastian Schaetz, Christophe Meyer, Abraham Neben, Bob Ryan, J. R. Petrus, Joe Lutsky, Dan McMahill, Gregory Corteville, Matthew R. Hageman, Larry Miller, Keith G. Fife

**Affiliations:** ^a^Butterfly Network, Inc., Guilford, CT 06437

**Keywords:** ultrasound, cardiology, semiconductors, global health, machine learning

## Abstract

Over the past half-century, ultrasound imaging has become a key technology for assessing an ever-widening range of medical conditions at all stages of life. Despite ultrasound’s proven value, expensive systems that require domain expertise in image acquisition and interpretation have limited its broad adoption. The proliferation of portable and low-cost ultrasound imaging can improve global health and also enable broad clinical and academic studies with great impact on the fields of medicine. Here, we describe the design of a complete ultrasound-on-chip, the first to be cleared by the Food and Drug Administration for 13 indications, comprising a two-dimensional array of silicon-based microelectromechanical systems (MEMS) ultrasonic sensors directly integrated into complementary metal–oxide–semiconductor-based control and processing electronics to enable an inexpensive whole-body imaging probe. The fabrication and design of the transducer array with on-chip analog and digital circuits, having an operating power consumption of 3 W or less, are described, in which approximately 9,000 seven-level feedback-based pulsers are individually addressable to each MEMS element and more than 11,000 amplifiers, more than 1,100 analog-to-digital converters, and more than 1 trillion operations per second are implemented. We quantify the measured performance and the ability to image areas of the body that traditionally takes three separate probes. Additionally, two applications of this platform are described—augmented reality assistance that guides the user in the acquisition of diagnostic-quality images of the heart and algorithms that automate the measurement of cardiac ejection fraction, an indicator of heart health.

## Significance

Affordable hand-held ultrasound is transforming health care as a diagnostic tool with the potential to be as ubiquitous as the stethoscope. Here, we present a platform for advancing diagnostic care consisting of an ultrasound-on-chip probe, leveraging state-of-the-art silicon-based semiconductor foundries, paired with a mobile device and artificial-intelligence–guided image interpretation and cloud interconnectivity. Demonstrations across key organs and modes illustrate the imaging capabilities. Presentations of automated guidance for untrained ultrasound users show the potential for further broadening accessibility and utility.

Current ultrasound systems are based on piezoelectric transducer technology (1–5). The ultrasound transducer is typically fabricated from a single piece of piezoelectric material, which is diced into individual elements. This process is laborious and costly, especially for fine-pitched and two-dimensional (2D) arrays. To interconnect the transducer to the electronics used for control and processing, shielded cables are generally connected to each element (often with significant additional manual labor). The number of such cables is often practically limited to a few hundred due to size and weight constraints, requiring the device to have an undesirably low channel count (often about 128 and well under 1,000). Moreover, since imaging at different depths in the body requires different ultrasound frequencies beyond what a single limited-bandwidth piezoelectric transducer can cover, piezoelectric probes must be specifically designed to serve only a subset of clinical applications. Generally, a minimum of three different classes of piezoelectric probes—linear, curvilinear, and sector/phased array—are required to cover the majority of indications needed for whole-body ultrasound scanning (6).

In order to address the shortcomings of piezoelectric technology, many academic and commercial attempts have been made to design micromachined electromechanical systems (MEMS) ultrasound transducers with varying success (7). Often, the performance achieved—e.g., bandwidth, pressure, and sensitivity—has not been sufficient for a wide range of clinical applications. Furthermore, creating a highly reliable and scalable MEMS transducer process has been elusively challenging (8, 9), especially in regard to achieving the substantial quality needed for medical device regulations (10, 11). Beyond MEMS processing, a complementary metal–oxide–semiconductor (CMOS) integration scheme solves the transducer-to-electronics interconnection problem, which is analogous to the solution of the circuit interconnection problem whose solution by Noyce (12) first birthed the semiconductor integrated circuit (IC) industry. Previous attempts to interconnect electronic control and signal processing for both analog and digital domains in ultrasound have been incomplete (13, 14), neither fully solving CMOS integration nor achieving functional clinical embodiments. State-of-the-art ultrasonic application-specific IC (ASIC) designs found in the literature have specifications as summarized in Table 1 (15–21).

**Table 1.** Comparison of this UoC to state-of-the-art ultrasound ASIC designs

		Ref.						
	This work	15	16	17	18	19	20	21
Process	130 nm BCD+MEMS	1.5 µm HV	28 nm	180 nm HV SOI	180 nm	180 nm	180 nm BCD SOI	350 nm CMUT-in-CMOS
Transducer	2D CMUT	2D CMUT	2D CMUT	2D PZT	2D PZT	2D PMUT	2D CMUT	2D CMUT
No. of TX/RX elements	8,960/8,960	256/256	0/16	128/3072	0/144	36/36	256/256	0/960
Operating frequency	1–10 MHz	5 MHz	5 MHz	5 MHz	5 MHz	5 MHz	7 MHz	5 MHz
Pitch-matched	✓ (208 µm)	×	✓ (250 µm)	✓ (300 µm)	✓ (150 µm)	✓ (250 µm)	✓ (220 µm)	✓ (162 µm)

Multilevel pulsing	✓ (7-level)	×	×	✓ (3-level)	×	×	×	×
TX beamforming	✓ (digital)	✓ (digital)	×	✓ (digital)	×	×	×	×
RX beamforming	✓ (digital)	×	✓ (digital)	✓ (analog)	✓ (analog)	✓ (analog)	×	×
On-chip TGC	✓ (0.2 dB/step)	×	✓ (0.33 dB/step)	×	✓ (6 dB/step)	✓ (6 dB/step)	×	✓ (4.3 dB/step)
On-chip ADC	✓ (1,120 ch)	×	✓ (16 ch)	×	✓ (16 ch)	✓ (36 ch)	×	×
On-chip DSP	✓	×	×	×	×	×	×	×

An ultrasound-on-chip (UoC) is described in the following sections that outline the design, fabrication and integration of MEMS ultrasonic transducers directly in a CMOS process. Furthermore, we show the detailed CMOS circuit designs that drive the MEMS and that accomplish data acquisition and processing. Together, the CMOS and MEMS constitute a UoC platform that enables a single-probe whole-body ultrasound imager, which achieves Food and Drug Association (FDA) clearance in 13 key indications (22)—more than any other single ultrasound probe prior. The mixed-signal (analog and digital) circuitry provides full processing and control for a versatile UoC platform. The supporting peripherals enable a suite of high-level applications having connectivity and upgradeability. The acoustic performance and a set of powerful imaging capabilities are shown. Finally, an artificial intelligence and augmented reality demonstration of automated guidance and interpretation shows the potential for expanding accessibility and utility.

## Results

**Fig. 1.**
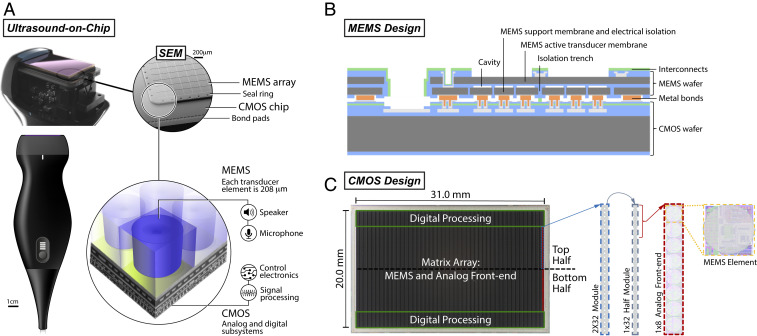
UoC design. (*A*) The 8,960 MEMS elements are on a single transducer-on-CMOS chip, each with control and processing circuitry for sending and receiving ultrasound signals through the acoustic lens of a handheld probe. The integrated MEMS and CMOS structures on a single die can be seen in a scanning electron microscope (SEM) image. The MEMS transducer array is bonded to the CMOS die and bounded by a seal ring. Bond pads to CMOS I/O connections are along the periphery of the CMOS chip. (*B*) Cross-section of the MEMS ultrasound transducer design. (*C*) A photograph of the UoC and modules replicated over the chip.

Fig. 1*A* shows a UoC probe form factor at multiple zoom scales where the 2D array of 140 × 64 (8,960) MEMS elements is directly integrated with the electronics on the CMOS without requiring any cable or wire interconnects (23). See *SI Appendix* for a flat profile form factor of a patch (*SI Appendix*, Fig. S1). MEMS membranes are coupled to CMOS electronics to enable transmission and reception of the ultrasound waves.

### UoC MEMS Design.

Fig. 1*B* shows wafer-level integration of the MEMS transducers onto CMOS circuits (24). The fabrication involves two separate wafer bonding steps: wafer bonding to fabricate sealed cavities in a substrate and wafer bonding between the MEMS substrate and the CMOS substrate. The former is accomplished with a high-temperature anneal, above 450 °C, to facilitate achieving a strong bond. The latter is performed with a low-temperature metal bond to maintain the integrity of the CMOS which has a thermal budget maximum of 450 °C.

The sealed cavities of the MEMs substrate are formed from bulk silicon (Si) and silicon-on-insulator (SOI) wafers. The SOI is highly doped for electrical conductivity of the membrane and an insulator is formed by thermally oxidizing the Si device layer. Cavities are formed in the thermal oxide. A highly doped bulk Si wafer is used to form the lower electrodes and isolation structures, patterned with trenches which electrically isolate individual elements. The wafers are bonded, and the bulk Si is thinned, leaving the handle layer of the SOI for mechanical integrity. After the low-temperature metal bond to the CMOS, the SOI handle layer is removed and interconnects are formed.

The active MEMS ultrasonic array is chosen to be about 30 mm × 13.3 mm with 140 × 64 elements, so as to accommodate a wide field of view. Each element is individually electrically connected and separately addressable from the CMOS. The element pitch, membrane thickness, and gap height are chosen to be 208 µm, 5 µm, and 200 nm, respectively, to support a broad ultrasound bandwidth of 1 MHz to 10 MHz. MEMS parameters were selected by combination of static analysis of plate theory and dynamic simulations using a one-dimensional equivalent circuit model (25, 26).

### UoC CMOS Design.

A 0.13-µm BCD (bipoloar-CMOS-double-diffused metal–oxide–semiconductor) chip, seen in Fig. 1*C*, is designed on a 31- × 20-mm die with the active MEMS array spanning over the analog front-end circuitry and the digital processing circuitry on the elevational periphery (27). Modularity of the design provides a reduction of the engineering complexity. A 2- × 32-element ultrasound processing unit (UPU) module is replicated 140 times across the top and bottom halves of the array. Each half-module has a digital processor and services a 1 × 32 column, which consists of four analog front ends each servicing eight MEMS elements. A modular communication protocol coupled with the modular physical design allows for scaling the array size with straightforward replication (28).

**Fig. 2.**
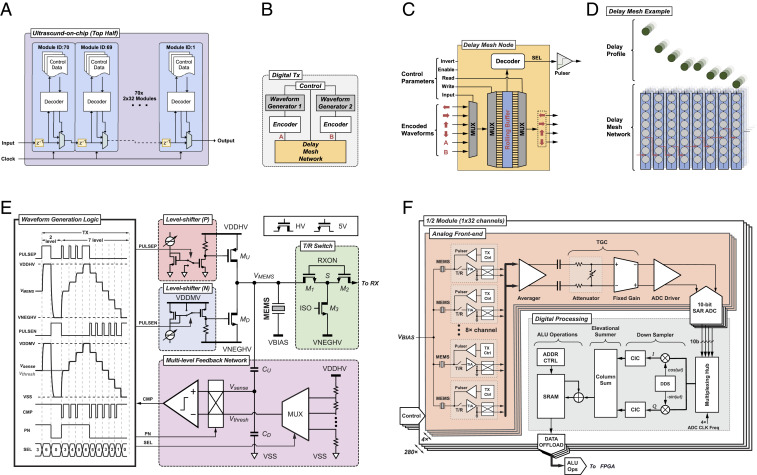
UoC schematics. (*A*) Module-level communication architecture. (*B*) Digital transmit waveform and delay controller. (*C*) Delay mesh node at each MEMS element. (*D*) Delay mesh network example delay profile. (*E*) Schematic of the multilevel pulser with tunable slew rate and its timing diagram. (*F*) Modular on-chip receiver design with analog circuitry supporting each group of eight elements in the analog front end. The transmit pulser and receive TIA circuitry are unique for each element. Element signals can be combined and share DC offset correction, dynamic TGC with arbitrary profile and analog-to-digital conversion (ADC) operations. Digital processing downstream of the ADCs performs baseband conversion for data reduction while preserving important signal content. Signals can be combined or separated in a variety of ways before being written to memory for offload.

Fig. 2*A* shows the digital communications subsystem between the 2- × 32-element UPU modules (29). The I/O interface of each module is simply one input port, one output port, and one clock. Parameter loading utilizes the module-to-module packet delivery where the destination is addressed by its module identification number. This allows packet communications to utilize local and global addressing in a low-latency, deterministic protocol. The UPU parameterization is modularized into categories to utilize symmetries of layout and global control packet communications, e.g. sequencer parameters, analog transmit parameters, analog receive parameters, time gain compensation (TGC) parameters, digital transmit parameters, waveform generator parameters, waveform delay parameters, or digital receiver parameters. Thus, a reduced number of control packets can be sent for identically configured UPU categories and updated only according to the changes between acquisitions.

The CMOS circuitry is designed to individually drive each MEMS element to transmit ultrasonic waves as well as to receive ultrasonic vibrations which are converted to electronic signals, amplified, and processed. The CMOS design is composed of mixed-signal subsystems which enable sophisticated control and signal chain processing. The following paragraphs describe the CMOS designs as one would follow the flow of a signal through the chip from waveform generation and pulsing for transmitting to receiver amplification and processing for imaging.

The on-chip digital transmit circuitry (Fig. 2*B*) enables each of the 8,960 elements to be individually addressed in transmit for full beam-forming capabilities in azimuth and elevation, which allows the transducer to have a programmable focal depth. A total of 280 waveform generators can be programmed with arbitrary waveforms having seven levels where each element can have independent delays at 6.25-ns increments (30). The arbitrary waveform generators enable long- and short-duration waveforms, such as pulses, chirps, coded excitations, sinusoids, square waves, pulse inversions, and apodized and phased waveforms to achieve a parameterized customized frequency response. Encoders and decoders allow for serialization at the system clock level for precise waveform delays (31). A delay mesh network provides the serialized link between elements for waveform distribution across the elements, yielding very deep cumulative delays with fine time resolution. Each delay mesh node (Fig. 2*C*) provides a programmable input source as well as a write and read selection for introducing a delay to the rolling buffered waveform. There are two waveform generators per UPU that can be injected at any delay mesh node. The architecture of this waveform distribution network allows for programming of complex waveforms, each capable of independent delay times for each element, as well as a method for distributing delays in separable manner between azimuth and elevation directions. Note that the acoustic lenses of traditional probes often differ based on the depth that they choose to optimally image. Here, the ability to set the elevational delays with the mesh parameters essentially enables any virtual acoustic lens on the UoC probe. The control parameters provide functions for waveform inversion as well as individually enabling the mesh and/or the SEL pulser driver signal. Thus, the aperture is completely configurable in shape and size for transmit (and similarly for receive). An illustrative example of the delay mesh network is shown in Fig. 2*D*, where the programmable buffer-to-buffer delays result in the delay profile. These arbitrary delay profiles provide the capability to electronically steer and focus the ultrasound beam in azimuth and elevation to enable multiprobe emulation for advanced 2D and three-dimensional (3D) imaging, e.g. virtual lenses, virtual sources, cylindrical waves, or plane waves.

A digitally controlled analog pulsing circuit is designed to provide a ±25-V swing in voltage potential at the MEMS electrode, *V*_*MEMS*_, while the voltage applied to the MEMS top membrane is fixed, VBIAS. A multilevel pulser architecture enables waveform shaping to optimize for the transfer function of the MEMS to improve contrast and linearity (32). Furthermore, it is capable of extended waveforms and chirps for use with matched filtering and pulse compression. Pulsing is achieved with a pair of pull–push high-voltage transistors (*M*_*U*_ and *M*_*D*_) that couple to each MEMS element, as shown in Fig. 2*E*. Multilevel pulsing is achieved with the aid of a feedback network. When operating in the multilevel mode, a high-voltage-tolerant voltage divider (*C*_*U*_ and *C*_*D*_) is used to scale down *V*_*MEMS*_ to the 5-V domain (*V*_*sense*_), where it is compared to a scaled-down version of the threshold voltage (*V*_*thresh*_) generated with a seven-tap resistor ladder. A dynamic control bit P/N sets the comparison polarity toward the target voltage, where the resulting comparator output signal (CMP) controls the activation duration of the pulser front-end (PULSEP and PULSEN). These digital feedback signals configure the HV pulser frontend *M*_*U*_ and *M*_*D*_ to either charge or discharge the MEMS capacitor until *V*_*sense*_ reaches *V*_*thresh*_, thus mastering the end voltage levels at *V*_*MEMS*_. Furthermore, a pair of current-controlled level shifters provide a variable overdrive voltage for *M*_*U*_ and *M*_*D*_, facilitating a tunable slew rate within pulsing frequencies from 1 MHz to 10 MHz (33). Both the pull-up and pull-down currents are controllable from 2.5 mA to 9 mA, corresponding to a slew rate ranging from 0.42 kV/μs to 1.5 kV/μs for a 6-pF load capacitance. This load capacitance consists of the electrical parasitic, less than 1 pF, and the MEMS capacitance, ∼5 pF. The power required to drive the MEMS transducer with the push–pull front end is dominated by the dynamic charging process, *P*_*pulser*_ = *fCp* (*VDDHV* − *VNEGHV*)^2^, where *f* is the pulsing frequency and *Cp* is the load capacitance. Since the pulsing duration time is only a fraction of the total receiving time (<∼1%), the pulser’s power consumption is not a dominant factor for the system power. Furthermore, the system is designed with high-voltage-tolerant bypass capacitors on the ASIC and the printed circuit board (PCB) as well as the power supply allow us to supply current across the 8,960-element array of pulsers without significant voltage droops, thus supporting extended waveforms, e.g. Doppler. In order to protect the low-voltage transistors in the receiver from the high-voltage transients of the transmitter, the T/R switch is designed with *M*_*1*_ and *M*_*2*_ having bulks tied to their sources (S). While pulsing, both the gates (RXON) and sources (S) of *M*_*1*_ and *M*_*2*_ are pulled down to the most negative voltage (VNEGHV). After pulsing, both *M*_*1*_ and *M*_*2*_ are turned on by pulling their gates, at RXON, up to 5 V, and optionally can be left floating during receive to reduce noise/interference coupling from 5-V supply to the sensitive signal path.

The UoC receive architecture is designed modularly with replicated arrays of pitch-matched analog front ends (AFEs) and digital processing units as seen in Fig. 2*F*. Each MEMS element interfaces with an independent area-matched ultrasound transceiver, consisting of the multilevel high-voltage (HV) pulser, the transmit (TX) waveform controller, the T/R switch, and a transimpedance amplifier (TIA) (34). A cross-coupled switch facilitates polarity changes on any of the differential pair input. Such a feature is helpful with coded aperture imaging modes, multiplexed Hadamard codes, or paired cancellation modes. The TIA is implemented as a two-stage Miller-compensated amplifier with a programmable feedback impedance ranging from 7.5 kΩ to 180 kΩ (35). The bias current in both stages of the amplifier can be digitally adjusted to meet diverse requirements for AFE noise and power in different imaging modes. With the AFE at its highest gain, we measured the channel noise at the analog-to-digital converter (ADC) output to be 8.68 mV rms and the channel interference noise also at the ADC output to be 0.55 mV rms, which is 24 dB lower than the channel noise floor. To enhance the signal-to-noise ratio, or to reduce the channel count, the outputs of one to eight TIAs in the same half-column (1 × 32) can be sum-and-averaged in the analog domain. Individually digitally configurable TIAs and averaging amplifiers provide the ability to average or multiplex any desired configuration of the eight channels. A 20-dB range 0.2-dB/step dynamic TGC with an arbitrary profile control is performed in the AFE to compensate for tissue attenuation (36). The TGC provides real-time shifting of the dynamic range of the receiver to properly sample signals whether strong from shallow acoustic reflection or weak from much deeper acoustic reflection. This is achieved by a voltage attenuator followed by a fixed-gain amplifier (FGA). The attenuator is implemented as a resistive divider, where the shunt resistor is programmable through 100 parallel legs. Each leg adds 0.2 dB of extra attenuation, leading to a maximum attenuation of −20 dB. The on/off states of resistor legs are controlled by a 100-bit shift register, where a logic “0” or “1” is serially shifted in synchronization with the ADC sampling for gain increment or decrement. The FGA provides a 20-dB fixed gain to match with the attenuation. It is implemented as a differential open-loop amplifier with resistive load. An output common-mode feedback circuit regulates the amplifier output to half VDD to facilitate driving the succeeding stage.

The FGA is followed by a wide-band pseudodifferential source follower that drives the on-chip ADC with a sampling rate up to 40 MHz. A 10-bit successive-approximation-register (SAR) ADC based on charge-redistribution and asynchronous switching is implemented in each AFE channel for local digitization (37). Particularly, a split capacitor digital-to-analog converter (CDAC) (38) is employed to reduce the input capacitance for better compactness. It adopts a monotonic capacitor switching sequence (39) to further reduce the total capacitance as well as the switching energy. The asynchronous operation eliminates the need for distributing high-speed internal clocks across the array, reducing power and noise. Furthermore, the architecture guarantees a signal-independent current drawn from the ADC references, which offers a critical advantage of minimizing cross-talk noise in an array with 1,120 ADCs. To gain a better conversion accuracy, several calibration techniques are applied to compensate for process mismatch. For example, several extra error-correction switch-capacitor stages are carefully added to the CDAC to mitigate the impact of comparator inaccuracy, trading for better ADC linearity and lower noise.

The digitized samples from every four ADCs are consumed by a digital processing block nominally operating at a 160-MHz system clock rate and are resynchronized to a parallel data bus with a multiplexing hub. Each channel is then heterodyned to baseband, low-pass filtered, and down-sampled to facilitate decimation for a data bandwidth reduction in the succeeding processing (40). The baseband operation is accomplished with a direct digital synthesizer where a local oscillator is configured to output two signals, 90° phase offset, which are multiplied with the channel to generate in-phase (*I*) and quadrature (*Q*) components. The *I*/*Q* complex data are then low-pass-filtered by configurable cascaded integrator-comb filters with down-sampling. A configurable elevational column summer adds the decimated data from the rows to beamform and further compress the data for storage and offload. The SRAM can store and retrieve data for use with arithmetic logic unit operations and subsequent excitations. The 1,120 ADCs on-chip can produce 448 Gbps of digitized ultrasound data and the 280 digital processing blocks provide an aggregate of over 1 trillion fixed-point operations per second.

The UoC probe contains the UoC board connected to both a main board and a power board with a battery. The main board contains a field programmable gate array (FPGA) and coordinates with the other boards and a universal serial bus (USB) interface to the mobile device. Additional peripherals on the boards monitor temperatures, currents, probe position and orientation, and more; see *Materials and Methods* for more details on the probe build.

The FPGA provides low-latency deterministic communication directly to the UoC platform and facilitates communication over the USB connection to a host mobile device. This architecture maximizes the data rates and minimizes the ultrasound pulse repetition intervals, thus improving imaging capabilities. The FPGA sets configuration parameters, triggers acquisitions, and offloads and processes data streams (41).

A sequence processing architecture and instruction set has been developed to coordinate imaging mode acquisition and processing (42). A sequence compiler on the host (the mobile device) compiles high-level imaging mode parameters into UoC parameters and a sequence command executable to be run on a sequence processing unit (SPU) on the FPGA. The SPU coordinates a real-time timing sequencer to load UoC parameters, launch triggers, and offload and process ultrasound data. Additional processing is architecturally reconfigurable and includes beamforming, compounding, and synthetic aperture imaging capabilities (43).

The UoC, FPGA, and mobile processing employ multistage delay-and-sum 2D and 3D beamforming useful for accommodating the different data rates at each stage (6). Coherent and noncoherent summation across the elements and different transmits are programmable between the UoC, the FPGA, and the mobile processor. Additionally, multiple digital filter stages provide antialiasing, noise removal, frequency compounding, and log detection. Postprocessing blocks provide scan conversion, incoherent spatial compounding, edge preserving filters, persistence, and color-scale remapping.

A mobile device application provides a touch-screen user interface to select preset modes and parameters for imaging that are compiled and communicated in real-time via the USB connection. The mobile device processors provide additional back-end processing and visualization capabilities to the ultrasound data stream. Measurement and annotation tools help assess the ultrasound captures. The mobile platform provides a means to categorize and share data in the cloud. Further 3D rendering is done on the mobile platform, in the cloud, or on a local computer.

### Experiments.

**Fig. 3.**
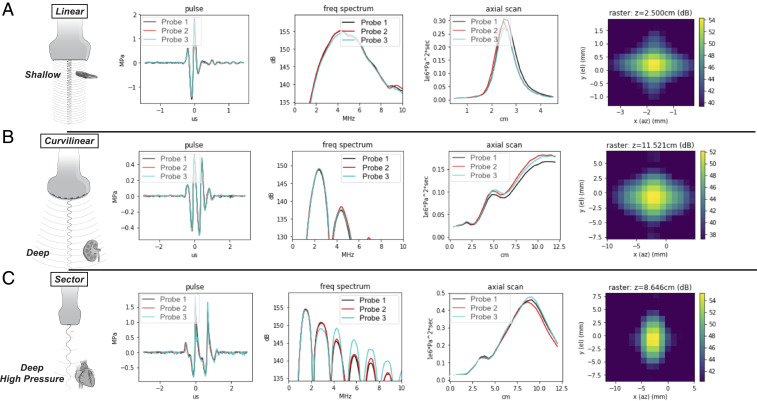
Universal probe acoustic verification measurements. The UoC probe is shown configured to operate in modes that have traditionally required three types of probes. The columns have focused beam measurements (temporal, spectral, axial, and lateral) from a water tank using three builds of the UoC probe, where the rows cover the three major categories of probe configurations. (*A*) A linear probe configuration suited for shallow imaging applications with higher frequency content and higher resolution, such as used for carotid artery imaging, where *S*_*r*_ is ∼200 μm. (*B*) A curvilinear probe configuration suited for middepth imaging applications where low-frequency pulses and a deep focus allow for applications such as abdominal imaging, where *S*_*r*_ is ∼550 μm. (*C*) A sector (or phased array) probe configuration suited for deep high-pressure imaging applications and harmonic generation for higher resolution, as used for cardiac harmonic imaging, where *S*_*r*_ is ∼650 μm.

The UoC is designed to operate over a range of 1 to 10 MHz, achieving the bandwidth, pressure, and sensitivity to image across the body. As such, the 208-µm pitch of the MEMS array provides suitable steering and focusing for low-frequency and broad-bandwidth probe emulation, respectively. The UoC has exceptional uniformity as measured across the array of 8,960 MEMS elements, where the SD of capacitance and gain is under 1 pF and 0.3 dB, respectively. Distribution plots of the measured characterization can be found in *SI Appendix*, Fig. S2. Fig. 3 *A*–*C* shows acoustic verification measurements corresponding to the responses for each emulation of linear, curvilinear, and sector probes each with three different UoC probes. The probe face is placed into a water tank and a submerged hydrophone (HNP-0200; Onda Corp.) collects pulse measurements at the focus and the focal plane and along an axial scan. Notice that the frequency response between all the emulated probes corresponds to desired applications of imaging shallow (broadband), deep (low frequency), or deep with high pressure (low frequency with harmonics). This is achieved by tuning the focus and waveforms using the digital transmit controller described above. The UoC probes have consistent measurements with each other, where the minor deviations in the higher harmonics of the sector scanning frequency response are a function of nonlinear harmonic generation. The image resolution has been measured with a performance of down to 200 µm both axial and laterally via in vitro testing. The quantitative relationship between each of the probe configurations and the achievable resolution, $S_{r}\geq c_{0}\tau /2$, can be directly calculated from the pulse width, *τ*, and the speed of sound, *c*_0_, as published by the World Health Organization (44), where the measurements in Fig. 3 comply with the International Electrotechnical Commission 62127 standard (45). For each of the probe configurations, the performance exceeds the resolution metrics set forth in the GB 10152-2009 standards and abides by the recommendations of the European Federation of Societies for Ultrasound in Medicine and Biology (46).

**Fig. 4.**
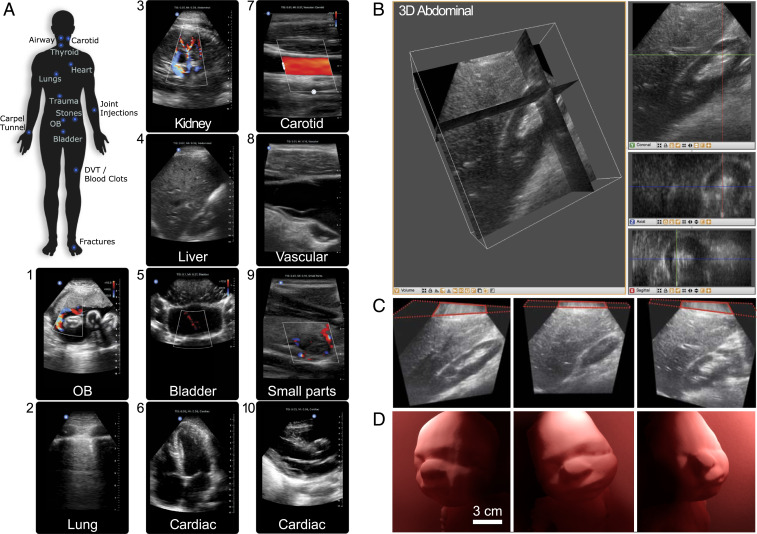
Ultrasound imaging with the UoC. (*A*) Labeled areas of the whole body where the UoC probe enables imaging. Example ultrasound imaging from several presets are labeled, including (1) OB: fetus umbilical cord Doppler flow; (2) lung: pleura interface; (3) abdominal: kidney vascular flow; (4) abdominal: liver; (5) pelvis: bladder jets; (6) cardiac: apical four chamber; (7) vascular: carotid artery; (8) vascular: internal jugular vein; (9) small organ: thyroid nodule; (10) cardiac: parasternal long axis. (*B*) Three-dimensional ultrasound renders of an in vivo human abdomen scan volume, 15 cm deep and 12 cm × 12 cm wide, where the structures and orientation between the kidney and liver can be viewed in multiple slices. (*Left*) Multiplanar reconstruction visualized in 3D with orthogonal slices and (*Right*) individual 2D image slices for coronal (XZ), axial (XY), and sagittal (YZ). (*C*, *Left*) Slice showing length of kidney and details of the renal cortex. (*C*, *Middle*) Slice showing the liver and edge of the kidney (Morison’s pouch). (*C*, *Right*) Slice showing liver parenchyma and kidney parenchyma. (*D*) Three-dimensional ultrasound of a 36-wk-old fetal ultrasound training phantom where surface rendering on a computer highlights contours of the baby’s face from different view angles. The ultrasound scan volume is 15 cm deep and 12 cm × 12 cm wide.

Broad probe versatility is a distinction of the integrated UoC. The traditional linear, curvilinear, and phased array probes needed for whole-body imaging can now be covered with a single ultrasound device. Common imaging locations cover the expanse of the human body as illustrated in Fig. 4*A* and demand specific features for optimal image quality. Structures in the neck, such as the airway, carotid, and thyroid, are optimized for shallow imaging with high-acoustic-frequency linear scanning. Superficial imaging of the arms, wrists, and varied joints is optimized by tuning with a dynamic aperture. This also allows transmit steering for optimized needle visualization during common invasive procedures. Abdominal imaging of kidneys, liver, bladder, or the pregnant uterus are configured for deep imaging with low acoustic frequencies for penetration and a wide field of view with steering for large cross-sectional area coverage. Imaging the heart, a dynamic organ, requires strict attention to pulse repetition intervals to ensure clarity and image quality. This is enhanced with harmonic imaging generating higher resolution and lower noise. Lung imaging for detecting pneumothorax (an abnormal collection of air in the pleural space between the lung and the chest wall) uses multiple optimizations to see B-lines at depth and to visualize lung sliding in shallow regions (47). Side-by-side image comparisons with predicate devices, spanning a representative set of indicated applications, have been evaluated by independent board-certified physicians for diagnostic equivalence (*SI Appendix*, Fig. S3).

Fig. 4*A* shows examples of 2D in vivo imaging across multiple body parts with the UoC probe, which is FDA-cleared for clinical indications of abdominal, cardiac adult, cardiac pediatric, carotid/arterial, fetal/obstetric, gynecological, musculoskeletal (conventional), musculoskeletal (superficial), pediatric, peripheral vessel, procedural guidance, small organ, and urology. Images and movies (Movies S1–S10) are provided for obstetric fetal umbilical cord Doppler blood flow, lung pleura boundaries, kidney low-flow perfusions deep within abdomen, liver hepatic vein structures near kidney, bladder jets with color Doppler showing ureters shooting into the bladder, cardiac apical four-chamber view, carotid artery with color Doppler showing high-flow-rate blood flow, vascular internal jugular vein, small parts thyroid nodule with low-flow color Doppler, and cardiac parasternal long-axis.

Beyond 2D ultrasound imaging for FDA-cleared indications which traditionally use three different types of piezo probes, more uses are enabled by the reprogrammable UoC sensor/processor. The vast flexibility incorporated into the UoC design required development forethought to enable state-of-the-art imaging modes, such as color and spectral Doppler, multiorgan, multiplane, and 3D imaging modes. Demonstration of spectral Doppler in the carotid artery with caliper measurements of peak systolic velocity, end diastolic velocity, and the time elapse (*SI Appendix*, Fig. S4 and Movie S11) showcases the high performance of UoC for high-speed acquisition (30+ kHz), long pulse durations (1+ μs), and beam steering (±30°). Fig. 4 *B* and *C* shows the 3D data of an in vivo abdominal scan where orientations show details of the renal cortex, liver, kidney, and Morison’s pouch; see Movie S12 for data elaboration. Fig. 4*D* shows a representative ultrasound volume collected on a 36-wk-old fetal ultrasound training phantom. For this data, the UoC platform was programmed to capture and process the 3D volume with a high fidelity at 1 volume per second. Such flexibility enables this programmable UoC platform to be a tool for extending ultrasound research and development of future modes tailored to specific clinical studies.

**Fig. 5.**
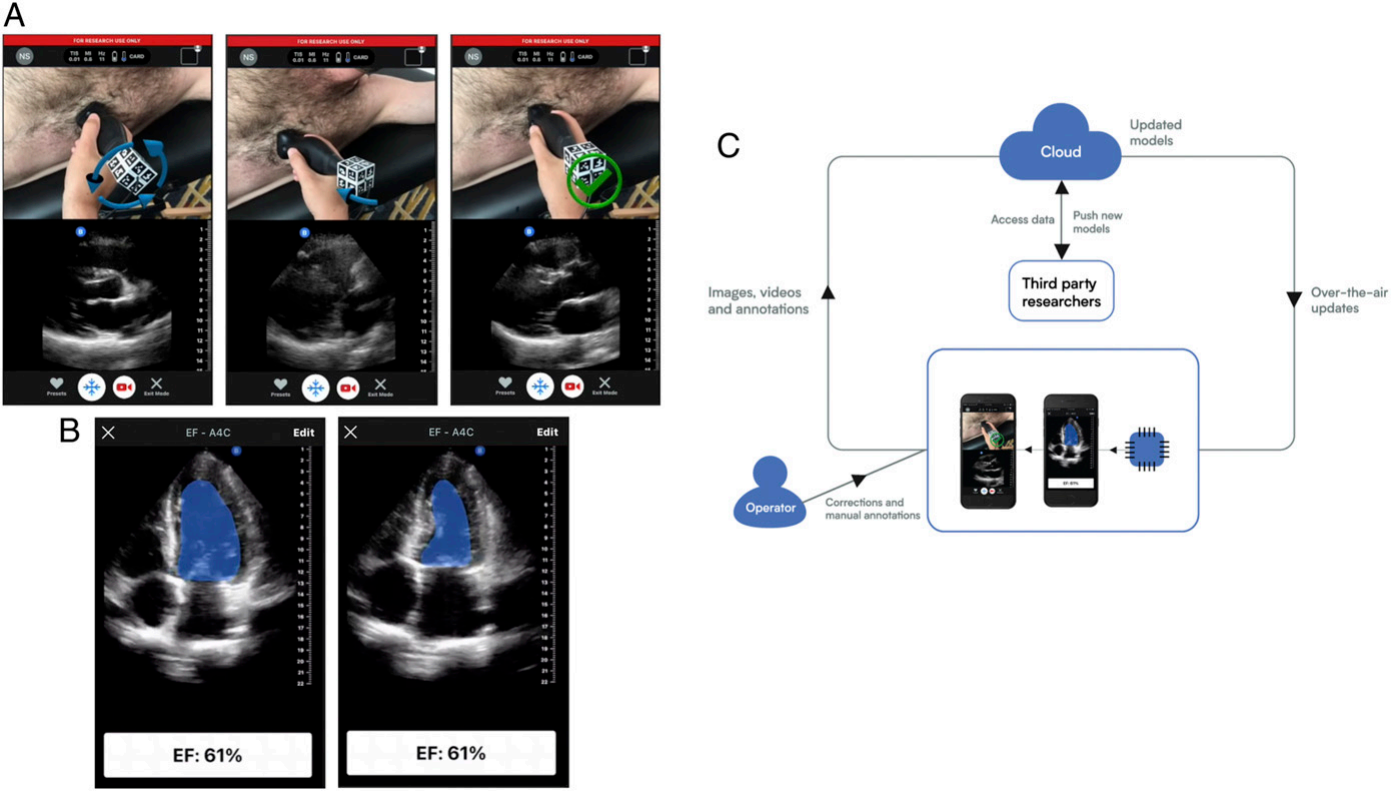
Forward-looking developments. (*A*) An assistance deep-learning model provides guidance to the user via a split screen where the camera image with augmented reality arrows overlain is shown on the top and the b-mode ultrasound image is shown on the bottom. Both frames are updated in real time. The screens show a cardiac scan where the arrows over the probe (*Left*) indicate a corrective action of counterclockwise rotation is needed, (*Middle*) indicates a corrective action of tilting the end of probe toward the subject’s feet is needed, and (*Right*) indicates that no corrective action is needed. (*B*) An interpretation deep-learning model is run on a captured movie of the heart beating and automates segmentation for the calculation of the EF of the heart by taking one minus the ratio of volumes of blood in the heart’s left ventricle (LV) at (*Left*) end systole and (*Right*) end diastole. The volume is calculated using a disc integration based on the cross-sectional area using a technique known as Simpson’s method (53). (*C*) A virtuous cycle in which ultrasound data are collected from devices in the field along with annotations, such as one might do in a research clinical study. These data are transmitted to a central location for analysis and to train models. The data annotations may be de novo or corrections to suggestions for a previous algorithm. The same data link that is used for retrieving data may also be used to deploy updated or new models. The cycle is further strengthened by leveraging acquisition assistance to guide a user to the correct acquisition and then assisting in the initial interpretation. As data are collected in the cloud, models improve by training on the correction data.

Expanding upon the capabilities provided by connecting a mobile device, we have designed an acquisition assistance application to interactively guide an operator to place and orient the UoC probe to a specified target anatomy. Convolutional neural networks consume the sequence of ultrasound images and produce movement instructions in real time. An augmented reality approach is used to convey the positioning instructions to the operator. A live video of the probe scanning is captured with the device’s camera and 3D arrows are rendered over the probe to intuitively convey the proposed movement in real time (Movie S13 and Fig. 5*A*). Once the operator acquires the target anatomical view or views as part of an ultrasound examination, anatomical structures may be traced and key points identified. In an effort to automate this process of interpretation for a common metric calculation of ejection fraction (EF), we developed an interpretation assistance feature, which calculates EF using two different methods, one by measuring cross-sectional area of the apical four-chamber view and the other by a linear measurement of the parasternal long axis view (Movie S14). Metrics are calculated using a ResNet architecture (48) integrated with an encoder–decoder path borrowed from a U-Net architecture (49). Annotation labels used for identifying keypoint locations of features, like boundaries and extents, are used to train the model. EF extent output keypoints, locations that the trained model identifies in an output result with sufficient confidence, are generated from a heat-map method (50) having a center of mass calculation (51). For the segmentation model, a threshold of the output heat map is taken for the largest connected region. A tracing of the segmentation border area is seen in Fig. 5*B*. The visualization builds user confidence for the calculation and offers an interactive user adjustment for the final calculation.

## Discussion

Generally, ultrasound systems are a shared resource among staff at a hospital and can require scheduling and time sharing to use effectively. When it comes to the utility of diagnostic ultrasound imaging, timing can be critically important, whether it is time to get feedback from medical professionals, time to load information from hospital records systems or store data to archives, time to control the device with its user interface, and even time to get the imaging device set up and usable. Saving time amounts to a quicker diagnosis, prognosis, and eventually recovery, where time can be of vital importance. The large capital expense of ultrasound systems usually prohibits people from having a personal ultrasound fully accessible, making cost a barrier to timeliness. It is widely recognized that building semiconductor chips leverages advantages of scale miniaturization and volume manufacturing to reduce production costs in complex circuitry. By integrating the circuitry of a full ultrasound system onto a chip, we hope that UoC enables a transformation of health care worldwide—just as putting a camera on a semiconductor chip (52) made photography accessible to anyone with a smartphone. A low-cost UoC device provides a means for distribution of a personal imaging system that is quickly accessible. A similar effect has been seen in the way smartphones have increased the personal accessibility and usage of telecommunications, computing, and picture and video generation.

Beyond offering high-quality medical imaging at a low price point for widespread adoption of point-of-care ultrasound, the UoC platform enables continued development of unique applications and form factors—including the low-profile ultrasound patch for patient monitoring (*SI Appendix*, Fig. S1). By combining the versatile UoC probe with a mobile device having broad interconnectedness, the applications for it become field-upgradable and integrated to the cloud and artificial intelligence systems. In order to open up the UoC platform for research and development of new applications, we are developing an API, SDK, and an applications repository to provide access to data aggregated in the cloud repository and to expose some of the hardware and processing interfaces. Understanding that the needs for ultrasound go beyond the probe hardware toward interconnectivity, our vision is to maintain a virtuous cycle framework, see Fig. 5*C*, that enables UoC data collection and clinical research and development for applications improving performance and the potential for automated interpretation.

## Materials and Methods

**Fig. 6.**
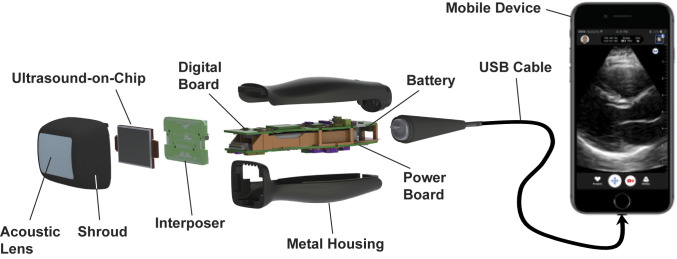
An exploded view of the UoC probe. The acoustic lens is held together with the UoC and interposer by a metal shroud. The shroud connects with the metal housing that contains a digital board, power board, and battery. The interposer board connects to the digital and power boards. The digital board and mechanical housing connect to a USB cable, which plugs into a mobile device for display.

Fig. 6 shows a breakdown of the UoC probe, where the MEMS and CMOS integrated chip is situated at the head of the probe. The chip’s front side is covered by the acoustic lens which is formed by room-temperature-vulcanizing silicone material. The back side is contacted by a copper-cladded aluminum nitride (AlN) heatsink, which is then coupled to the probe's metal shroud and metal housing. The UoC I/O pins are wire-bonded to a PCB interposer, which is plugged into the probe’s digital and power boards. The digital board contains an FPGA (Cyclone V; Intel Co.), which is responsible for initializing, programming, and controlling the UoC, as well as offloading ultrasound data from the UoC for further processing. The processed ultrasound data are then transmitted through the USB interface, a USB controlling chip (FX3; Cypress Semiconductor Corp.), to the mobile device for image display. An authentication chip provides platform access (MFi MCCATCPZ003-MXHDM-T; Apple Inc.). The power board contains an eight-channel power management chip (LTC2977; Linear Technology) and a set of switching direct current (DC)–DC power converters that generate all voltage rails needed by the UoC and the digital board. Its power source is a 2,600-mAh lithium-ion battery, which can be wirelessly charged through the Qi wireless charging standard and managed by the power management chip (MAX77818; Maxim Integrated). In general, imaging modes are designed to run with a total power consumption of less than 5 W. About 50% of the power is consumed by the UoC, while the signal processing in the FPGA demands another 30%. The remaining 20% is used by the power management units and miscellaneous components in the probe. On average, this guarantees more than two continuous hours of scanning and over 1.5 continuous hours when using higher-power modes like color flow imaging. Furthermore, the temperature at the probe head is monitored during operation to keep its heat from exceeding 43 °C, the point at which the probe is automatically powered down. During room temperature use, this only happens when scanning in the highest power modes for over 20 min.

This research study has been approved by the New England Institutional Review Board and allows for capturing image data with the device for research and development purposes. The investigators obtained the approved informed consent documents from all subjects prior to their enrollment in the research study.

## Supplementary Material

Supplementary File

Supplementary File

Supplementary File

Supplementary File

Supplementary File

Supplementary File

Supplementary File

Supplementary File

Supplementary File

Supplementary File

Supplementary File

Supplementary File

Supplementary File

Supplementary File

Supplementary File

## Data Availability

There are no data underlying this work.
