# Supplementary material for: Ultrasound-on-chip platform for medical imaging, analysis, and collective intelligence
Source: Proc Natl Acad Sci U S A. 2021 Jul 1;118(27):e2019339118. doi: 10.1073/pnas.2019339118 (PMC8271708; doi:10.1073/pnas.2019339118)
Supplement: Supplementary File [file pnas.2019339118.sapp.pdf]

PNAS
www.pnas.org

Supplementary Information for

Ultrasound-on-chip platform for medical imaging, analysis, and collective intelligence

Jonathan M. Rothberg*[1], Tyler S. Ralston[1], Alex G. Rothberg[1], John Martin[1], Jaime S. Zahorian[1], Susan A. Alie[1], Nevada J. Sanchez[1], Kailiang Chen[1], Chao Chen[1],Karl Thiele[1], David Grosjean[1], Jungwook Yang[1], Liewei Bao[1], Rob Schneider[1], Sebastian Schaetz[1], Christophe Meyer[1], Abraham Neben[1], Bob Ryan[1], J.R. Petrus[1], Joe Lutsky[1], Dan McMahill[1], Gregory Corteville[1], Matthew R. Hageman[1], Larry Miller[1], Keith G. Fife[1]
[1]Butterfly Network, Inc.

*Jonathan M. Rothberg, Tyler S. Ralston
Email: jonathan.rothberg@gmail.com, tyler.ralston@gmail.com

This PDF file includes:

Supplementary text
Figures S1 to S4
Table S1
Legends for Movies S1 to S14
SI References

Other supplementary materials for this manuscript include the following:

Movies S1 to S14

Supplementary Information Text

Artificial Intelligence Studies

Augmented Reality. The acquisition assistance system ingests data in the reference frame of the probe (the B-mode ultrasound data) and produces output in the reference frame of the operator's phone for the augmented reality overlay. The frame of the probe is estimated in reference to the subject. We assume that the operator and phone reference frames are the same. In order to calculate the transformation from the probe reference frame to the phone reference frame, we use the camera on the phone and an ArUco marker *(1)* attached at a known location and orientation to the probe. Research shows that the ArUco marker pose accuracy achieved an average max jitter of under 1-degree rotation error. *(2)*. We use OpenCV (https://docs.opencv.org/3.1.0/d5/dae/tutorial_aruco_detection.html) to perform the calculations. The location and orientation data are merged with the instruction produced by the acquisition assistance and then the directions are rendered on screen using SceneKit (https://developer.apple.com/documentation/scenekit).

Acquisition Assistance – Experimental data collection, algorithm training and evaluation. In order to train the acquisition assistance (AA) model, multiple operators acquire sequences of 2D ultrasound data from a number of subjects. The operator follows an on-screen guided acquisition protocol.

We employ a hierarchy of techniques in order to evaluate the performance of the acquisition assistance system. Some of these techniques have a lower fidelity score relative to the real system but allow for easy to calculate metrics. We refer to these techniques as *in silico* scan simulations. Otherwise, *in vivo* techniques are employed to replicate the system in clinical settings. We can measure success in the *in vivo* experiments as either successfully measuring the target anatomy, or more simply as successfully reaching a target point (as determined by a human expert). We employed other techniques for improving the fidelity of the simulation through hard example mining *(3)* and data augmentation *(4)*.

In our *in silico* scans, we observe an overall per frame classification accuracy of 59%, where the classification is the direction that the probe should be moved. Note that this includes frames that are not categorized with any direction (not meeting threshold value) and those frames that need to move in two direction categories with different magnitudes, e.g. a mixture of down and right for a diagonal movement. A “flicker” is a case in which in the stream of instructions for the operator there is a transition from a correct instruction to an incorrect one. A multi-frame voting scheme helps to reduce or remove the errant flickers, thus providing a stream of positioning commands to the operator with higher fidelity having on average only 2.4 flicker frames per cine (each on average having 110 frames).

In order to evaluate the acquisition assistance model, we recruited 58 volunteer scanners with no clinical experience. Using a time limit of 60 seconds, 54 of 58 scanners were able to use AA to navigate to a PLAX view as defined by agreement between the model and the clinician, see Figure 5A for an example. Based on this pilot study as proof-of-concept, further studies are now being conducted for full clinical validation with more volunteer scanners; a large group of subjects with a range of size, weight, and heart health assessments; in both PLAX and apical 4 chamber views.

Interpretation Assistance – Experimental data collection, algorithm training and evaluation. The training data sets for interpretation assistance were collected from expert sonographers imaging the heart from an apical four-chamber view and a parasternal long axis view from a variety of subjects. Interpretation experts reviewed and segmented the ultrasound images by hand from the collected data sets. The CNN model is trained and validated on 4-5 cines per view of 200 subjects with 8 operators with 5 labelling experts in total. The training and validation sets had completely disjoint sets of labelers, subjects, and operators. In this pilot study, the labelling experts are chosen from a different set of people than the operators collecting the data. All of the scanning subjects are also independent participants. This helps diversify the data collection and measurements. Additional clinical validation studies are in progress with a broader group of subjects having characteristics diverse in age, sex, weight, and heart health.

Expert sonographers have experience finding the proper scan plane of the heart where the chambers align with the apical view for clarity of the four chambers. The anatomy of the heart as seen in the scan plane provides clear boundaries for correct image plane placement. That being said, even between experts there may be slight deviations in placement. We mitigate any slight scanning plane deviations between scans by having multiple operators image the same subjects and comparing for concordance among operators and subjects *(5)*. The degree of acceptable metric accuracy is built into the acquisition assistance model.

The traditional methods of ejection fraction (EF) are calculated with either an area measurement or a linear measurement. The area measurements use the ultrasound of an apical 2- or 4-chamber view in conjunction with the Monoplane Simpson's method of calculation. Similarly, the linear measurement of the left ventricle diameter is performed using the ultrasound of a parasternal long-axis view (PLAX) in conjunction with the Teichholz method of calculation. Table S1 compares agreement of EF measurements between human-to-human as opposed to algorithm-to-human. Our experiments show an average of 61.3% human-to-human concordance and 64.3% algorithm-to-human concordance for area measurements of EF vs. 74% human-to-human concordance and 68.3% algorithm-to-human concordance for linear measurements of EF. The variability of the 1D measurements in the PLAX view results in an algorithm-to-human concordance value that is lower as a result of broader mean estimation than the localized human-to-human measurements. The concordance correlation coefficient is computed on the N-length measurements (i.e., N paired measurement values $(x_n, y_n)(x_n, y_n)$, for $n = 1, ..., Nn = 1, ..., N$) with the following formula:

$$\hat{\rho}_c = \frac{2s_{xy}}{s_x^2 + s_y^2 + (\bar{x} - \bar{y})^2},$$

where the mean is $\bar{x} = 1/N \sum_{n=1}^{N} x_n$, the variance is $s_x^2 = 1/N \sum_{n=1}^{N} (x_n - \bar{x})^2$, and the covariance is $s_{xy} = 1/N \sum_{n=1}^{N} (x_n - \bar{x})(y_n - \bar{y})$.

Furthermore, leveraging the training of scanning plane deviations from expert scanning and labelling, we have implemented a red-to-green meter indicating how close the operator is to an accurate scanning plane for chamber area calculations. This red-to-green meter complements the augmented reality probe placement directions.

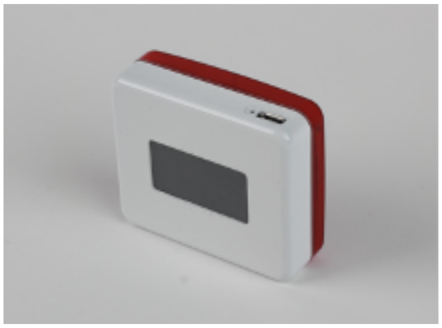

Fig. S1. Ultrasound-on-chip patch prototype. A functioning prototype of an ultrasound “patch,” build using Ultrasound-on-Chip technology.

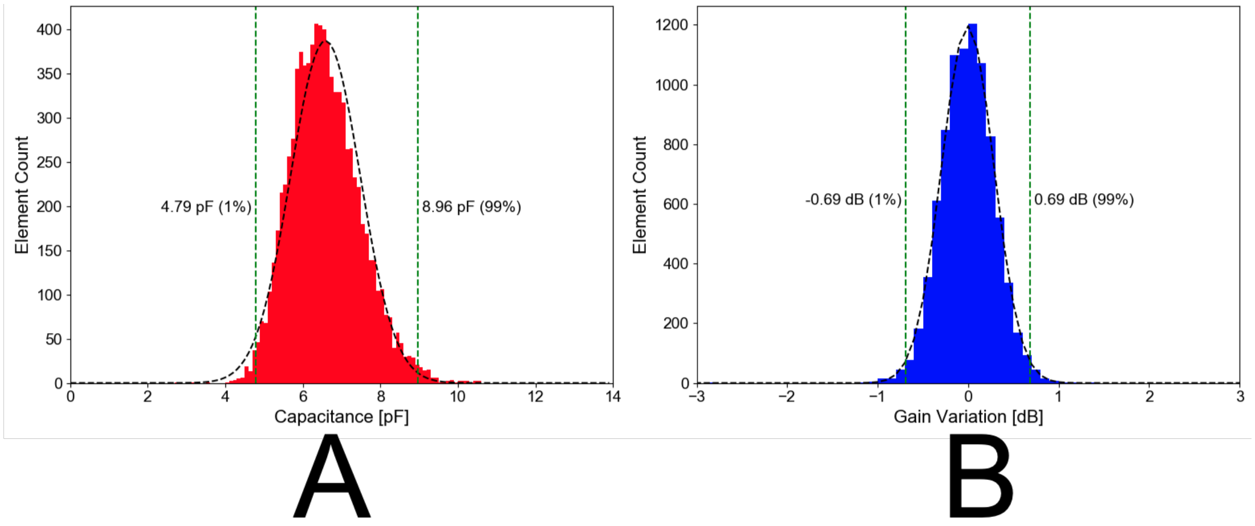

Fig. S2. Measured MEMS Element Characterization. (A) Capacitance, (B) Pulse-Echo Roundtrip Gain Variation.

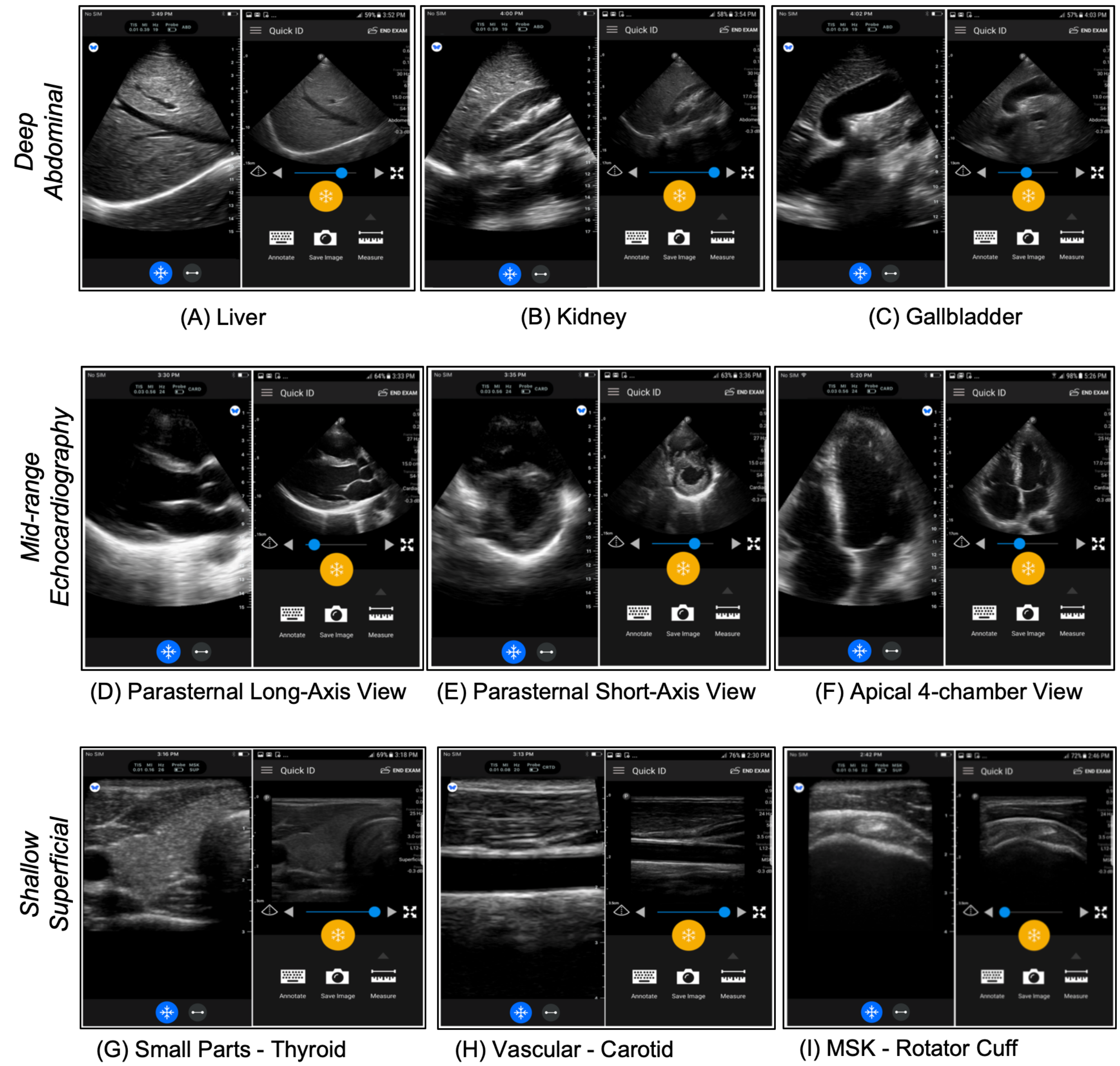

Fig. S3. Pre-FDA submission side-by-side comparison with predicate device. On-screen image pairs are shown between the ultrasound-on-chip device (left) and a corresponding scan using a Lumify device (right). Each device is configured to operate in 3 imaging modes:
Deep abdominal (top row): (A) liver, (B), kidney, (C) gallbladder Mid-range echocardiography (middle row): (D) parasternal long-axis, (E) parasternal short-axis, (F) apical 4-chamber Shallow superficial (bottom row): (G) thyroid, (H) carotid, (I) rotator cuff

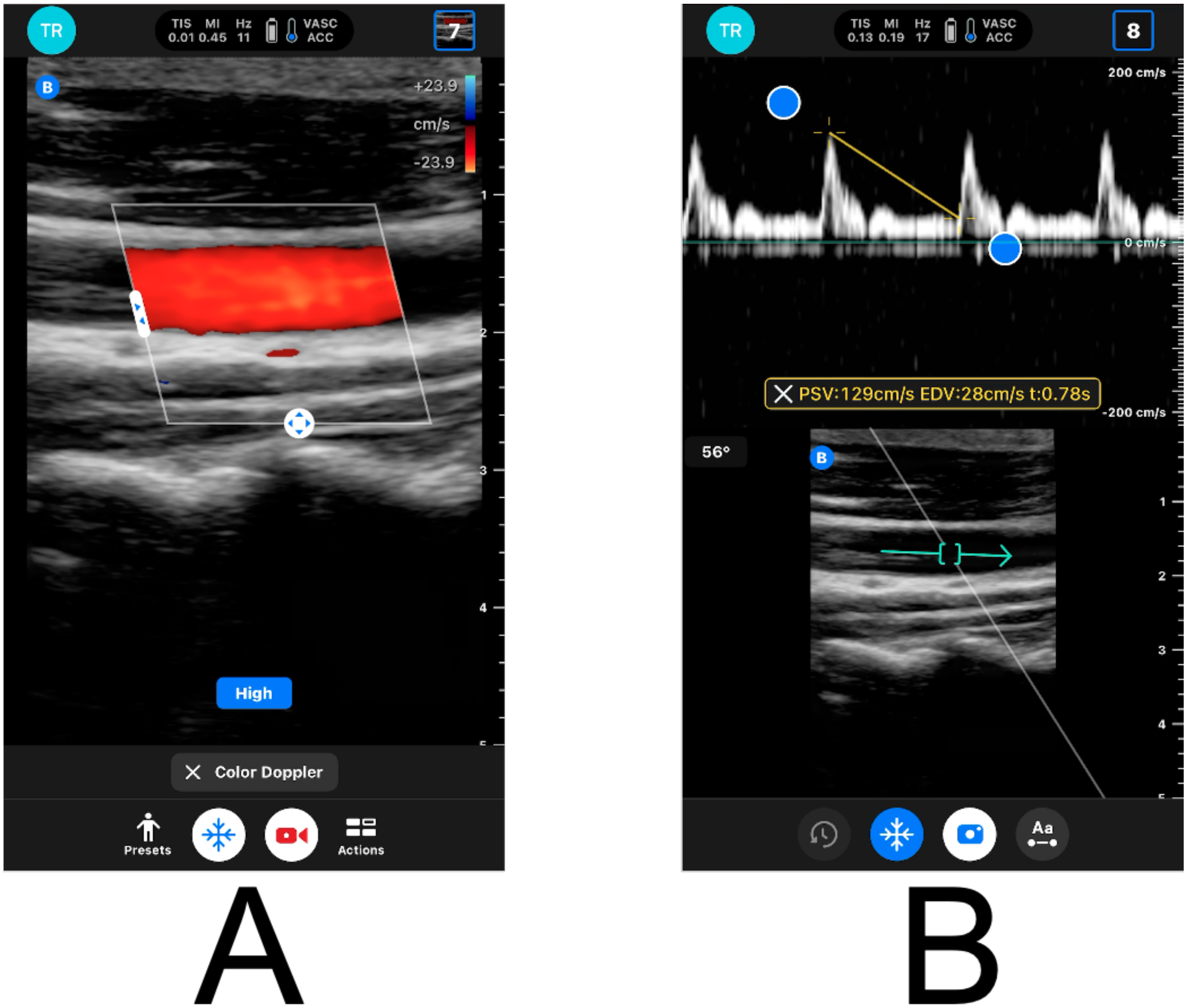

Fig. S4. Ultrasound-on-chip Doppler imaging of the carotid artery. (A) Color Doppler flow (B) Spectral Doppler flow calculation using an adjustable gate and angle overlay on the B-mode image. The calipers measure a peak systolic velocity (PSV), end diastolic velocity (EDV), and the time elapsed.

Apical 2- and 4-Chamber View (Simpson's method)(6) (area measurement)

Cardiac Ejection Fraction Apical 2- and 4-Chamber View	Concordance Correlation Coefficient *(5)* (higher better)
**Average Human-to-Human**	**61.3% ± 8.2 %**
**Average Machine-to-Human**	**64.3% ± 4.9%**

PLAX View (Teichholz method)(7) (linear measurement)

Cardiac Ejection Fraction Parasternal Long-Axis View	Concordance Correlation Coefficient (higher better)
**Average Human-to-Human**	**74.0% ± 3.6%**
**Average Machine-to-Human**	**68.3% ± 10.0%**

Table S1 Results from interpretation assistance models. We evaluate our models with two different methods of calculating Ejection Fraction: a segmentation of the LV chamber and a linear measurement of the LV diameter. Concordance was calculated between pairs of annotators (machine or human) who both annotated no less than 20 of the same images (classifying them in one of four diagnostic ranges) *(8)*. The concordance results indicate that our approach exceeds human-level performance on the area measurement and generalizes to the linear measurement.

Movie S1 OB: fetus umbilical cord Doppler flow

Movie S2 Lung: pleura interface

Movie S3 Abdominal: Kidney Vascular Flow

Movie S4 Abdominal: Liver

Movie S5 Pelvis: Bladder Jets

Movie S6 Cardiac: Apical 4 Chamber

Movie S7 Vascular: Carotid Artery

Movie S8 Vascular: Internal Jugular Vein

Movie S9 Small Organ: Thyroid Nodule

Movie S10 Cardiac: Parasternal Long Axis

Movie S11 Vascular Access: Carotid Artery Spectral Doppler

Movie S12 3D Ultrasound of Human Kidney in vivo

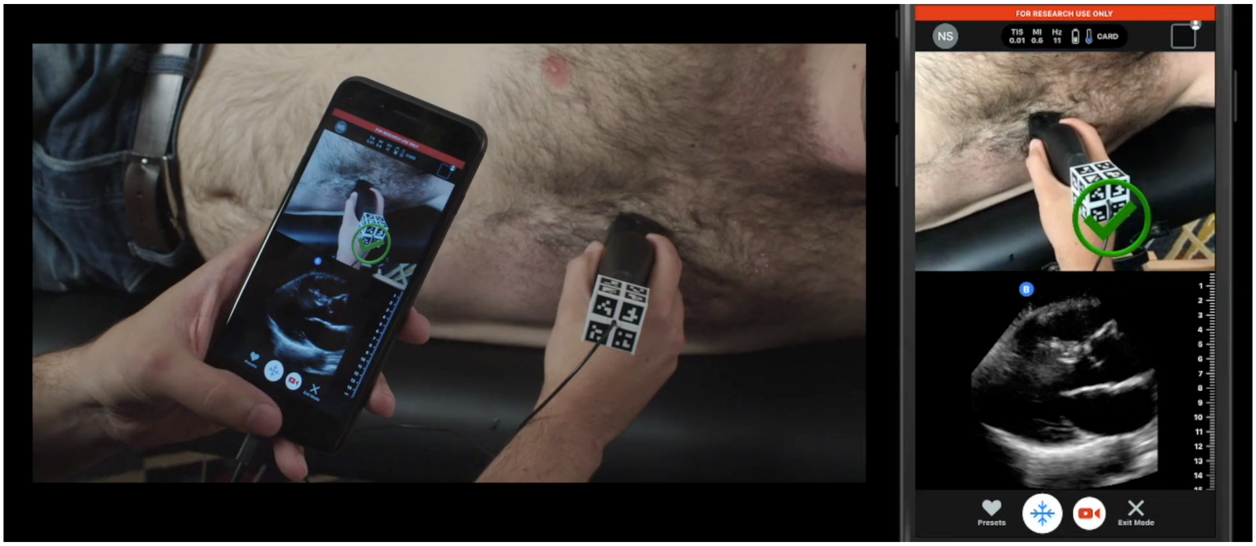

Movie S13. Acquisition Assistance.

Supplementary Movie 13 shows a demonstration of the interpretation assistance which guides the user toward the correct location by providing a quality indicator at the bottom of the screen. After the user achieves a high-quality orientation by the indicator, a video clip can be collected which is then used for an ejection fraction computation from diastole and systole segmentation of distances achieved between the septum and ventricular walls, a result of the thresholded heatmap described above. Similar acquisition is performed with an Apical Four Chamber view of the heart and where a segmented chamber is used to calculate an estimate for the ejection fraction and relay a quality score.

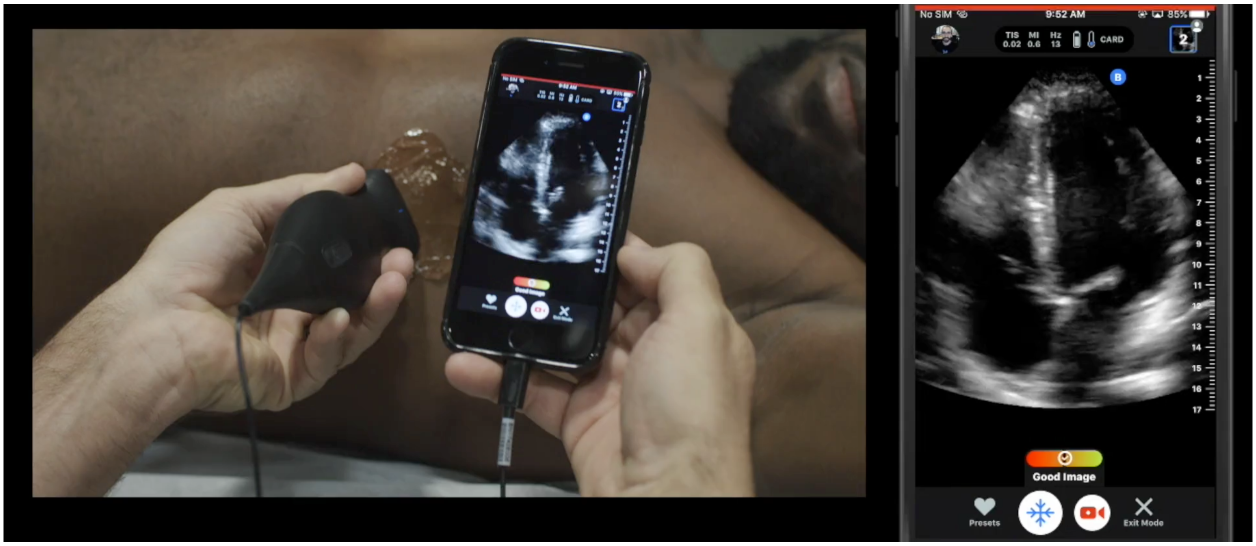

Movie S14. Interpretation Assistance.

Supplementary Movie 14 shows a demonstration of the acquisition assistance where the user aims the mobile phone camera to view the probe scanning. A split screen shows the ultrasound on the bottom and the camera output on top shows the probe with arrow overlays guiding the user to place the probe in the proper location for a cardiac exam, parasternal long axis (PLAX) view. A green check mark indicates the proper position.

SI References

1. Garrido-Jurado, S., Muñoz-Salinas, R., Madrid-Cuevas, F.J. and Marín-Jiménez, M.J., 2014. Automatic generation and detection of highly reliable fiducial markers under occlusion. Pattern Recognition, 47(6), pp.2280-2292.
2. Yu, G., Hu, Y. and Dai, J., 2019. Topotag: A robust and scalable topological fiducial marker system. arXiv preprint arXiv:1908.01450.
3. Zhao, D. and Peng, H., 2017. From the lab to the street: Solving the challenge of accelerating automated vehicle testing. arXiv preprint arXiv:1707.04792.
4. Tian, Y., Pei, K., Jana, S. and Ray, B., 2018, May. Deeptest: Automated testing of deep-neural-network-driven autonomous cars. In Proceedings of the 40th international conference on software engineering (pp. 303-314).
5. Lawrence I-Kuei Lin (March 1989). "A concordance correlation coefficient to evaluate reproducibility". Biometrics. International Biometric Society. 45 (1): 255–268.
6. Schiller NB, Shah PM, Crawford M et al. Recommendations for quantitation of the left ventricle by two-dimensional echocardiography. Am J Soc. Echocard 1989; 2: 358-68.
7. Teichholz LE, Kreulen T, Herman MV, et al. Problems in echocardiographic volume determinations: echocardiographic angiographic correlations in the presence or absence of asynergy. Am J Cardiol 1976; 37:7–11.
8. Lang, R.M., Badano, L.P., Mor-Avi, V., Afilalo, J., Armstrong, A., Ernande, L., Flachskampf, F.A., Foster, E., Goldstein, S.A., Kuznetsova, T. and Lancellotti, P., 2015. Recommendations for cardiac chamber quantification by echocardiography in adults: an update from the American Society of Echocardiography and the European Association of Cardiovascular Imaging. European Heart Journal-Cardiovascular Imaging, 16(3), pp.233-271.
